# An update on immune-based alpha-synuclein trials in Parkinson’s disease

**DOI:** 10.1007/s00415-024-12770-x

**Published:** 2024-12-12

**Authors:** Maha Alfaidi, Roger A. Barker, Wei-Li Kuan

**Affiliations:** 1 John Van Geest Centre for Brain Repair and Department of Clinical Neuroscience, University of Cambridge, Cambridge, CB2 0PY UK; 2 Department of Neurology, Addenbrooke’s Hospital, Cambridge, CB2 0QQ UK; 3 Wellcome Trust- MRC Cambridge Stem Cell Centre, Cambridge, CB2 1QR UK; 4 ALBORADA Drug Discovery Institute, Cambridge, CB2 0AH UK

**Keywords:** α-Synuclein (α-syn), Parkinson’s disease (PD), Clinical trials, Immune therapy, Neurodegeneration

## Abstract

Parkinson’s disease (PD) is a prevalent, chronic neurodegenerative disorder characterised by the progressive loss of dopaminergic neurons in the substantia nigra and other brain regions. The aggregation of alpha-synuclein (α-syn) into Lewy bodies and neurites is a key pathological feature associated with PD and its progression. Many therapeutic studies aim to target these aggregated forms of α-syn to potentially slow down or stop disease progression in PD. This review provides a comprehensive analysis of recent clinical trials involving vaccines and monoclonal antibodies targeting α-syn. Specifically, UB-312, AFFITOPE PD01A, PD03A and ACI-7104.056 are designed to provoke an immune response against α-syn (active immunisation), while Prasinezumab and Cinpanemab, MEDI1341 and Lu AF82422 focus on directly targeting α-syn aggregates (passive immunisation). Despite some promising results, challenges such as variable efficacy and trial discontinuations persist. Future research must address these challenges to advance disease-modifying therapies for PD around this therapeutic target.

## Introduction

Parkinson’s disease (PD) is a progressive neurodegenerative disorder that has a range of both motor and non-motor features. The motor symptoms and signs include resting tremor, muscle rigidity, and bradykinesia, along with gait and bulbar problems. While tremor and rigidity are also common symptoms, bradykinesia is considered a key marker of disease progression and severity and often serves as one of the earliest indicators of the condition. In addition to these, the patient experiences many non-motor complications, such as cognitive impairment, including dementia, affective disturbances, sleep problems, fatigue, constipation and a range of autonomic problems [1, 2]. This range of features maps onto a pathology that extends across many brain regions as well as in the periphery, such as the enteric and autonomic nervous systems. Currently, the mainstay of therapy is around replacing the dopaminergic nigrostriatal loss with drugs that work on this system. While effective symptomatically, especially in early-stage disease, they start to fail and produce their own side effects, and the disease progresses. Although other drugs and therapies are used to treat this and other aspects of PD, there are currently no disease-modifying therapies for it. To date, physical activity remains the only approach with evidence supporting its disease-modifying effects in PD, demonstrating its ability to slow disease progression [3].

PD is characterised pathologically by the accumulation of intracellular inclusion bodies, known as Lewy bodies (LB) and Lewy neurites (LN) [4], composed of misfolded alpha-synuclein (α-syn) [5]. In PD, α-syn misfolds and aggregates, leading to the formation of these inclusions, which initially disrupt neuronal function [6–8]. Although the pathogenesis of PD is not fully elucidated, it is evident that α-syn plays a pivotal role. This association was initially proposed following the major finding in 1997 of mutations in the *SNCA* gene in a familial autosomal dominant PD [9]. Subsequently, α-syn was recognised as one of the main components of LB and LN [5], suggesting their involvement in the pathogenesis of PD [10]. Importantly, α-syn aggregation is also implicated in related neurodegenerative disorders like dementia with Lewy bodies (DLB) and Multiple System Atrophy (MSA) [11, 12]. In addition, there is much interest not only in this cell-autonomous function of α-syn in PD but also in a non-cell autonomous process whereby α-syn can spread across neural networks, causing disease in a prion-like fashion [13]. All of this has meant that α-syn has become a major therapeutic target in PD, with one aim, in particular, being to stop the production and spread of the pathological form of α-syn through an immunisation approach and, by so doing, slow down the condition. In this update, we will present and discuss where we are with these trials currently (Table 1).

**Table 1:** Summary of recent clinical trials targeting alpha-synuclein immunologically

Drug Name	Sponsor	Modality	Status	ClinicalTrials.gov Number
UB-312	Vaxxinity, Inc	Vaccine	Phase I-completed Phase Ib-active	NCT04075318 NCT05634876
AFFITOPE PD01A,	AFFiRiS	Vaccine	Phase I-completed Phase I-completed Phase I-completed Phase I-completed Phase I-withdrawn	NCT02618941 NCT02216188 NCT01568099 NCT01885494 NCT02758730
PD03A	AFFiRiS	Vaccine	Phase I-completed	NCT02267434
ACI-7104.056	AC Immune	Vaccine	Phase II-recruiting	NCT06015841
Prasinezumab	Hoffmann-La Roche	Antibody	Phase I-completed Phase I-completed Phase II-active Phase IIb-active	NCT02095171 NCT02157714 NCT03100149 NCT04777331
Cinpanemab	Biogen	Antibody	Phase I-completed Phase I-terminated Phase II-terminated	NCT02459886 NCT03716570 NCT03318523
MEDI1341	AstraZeneca	Antibody	Phase I-completed Phase I-completed	NCT03272165 NCT04449484
Lu AF82422	Lundbeck A/S	Antibody	Phase I-completed Phase I-active	NCT03611569 NCT06258720

## Immunotherapy targeting alpha-synuclein

### UB-312

UB-312 is a synthetic peptide vaccine developed by Vaxxinity to target the insoluble α-syn protein. The active vaccine generates robust and time-dependent serum antibodies against the C-terminal epitope of α-syn 97–135, with titres peaking at week 29 and remaining above baseline values at week 45 [14].

A Phase I clinical trial (NCT04075318) was conducted to assess the safety, tolerability, and immunogenicity of the PD immunotherapeutic vaccine UB-312 in both healthy participants and those with PD. The study consisted of two parts: Part A involved escalating doses of UB-312, which were tested against placebo in healthy participants (*n* = 6), while Part B included two cohorts of participants with PD receiving different dosing regimens (*n* = 7, 300 μg /100 μg /100 μg); (*n* = 7, 300 μg /300 μg /300 μg) [14]. The most reported treatment-emergent adverse events were headache, local pain after lumbar puncture, and fatigue, with similar frequencies in the UB-312 (14 of 14 participants) and placebo groups (5 of 6 participants). Most treatment-emergent adverse events were mild or moderate, comparable to the placebo group. Three serious adverse events were reported, one of which—a deep venous thrombosis in the left leg occurring 50 days after the second administration of UB-312 at a dose of 300/100/100 μg [14]. No other safety concerns were identified on electrocardiogram (ECG) or with vital signs and blood and urine analyses [14]. The study’s primary outcomes focused on safety, tolerability, and immunogenicity. Two exploratory cerebrospinal fluid (CSF) biomarkers have shown potential as indicators of target engagement: aggregated α-syn, measured using a seeding amplification assay (SAA), and phosphorylated α-syn (pS129- α-syn). Exploratory analysis showed no statistically significant differences in clinical scales [14]. However, in PD patients, those with UB-312-induced antibodies present in the CSF exhibited significantly lower levels of α-syn aggregation (*p* < 0.01) and pS129-α-syn (*p* < 0.05) compared to patients who did not have detectable antibody titres in their CSF [14]. These findings were sufficient for the company to support the continued development of UB-312 in a clinical trial.

A Phase Ib clinical trial (NCT05634876) began recently and is in progress. Four patients with MSA and four with PD are being administered a total of eight doses of 300 μg UB-312 over two years, consisting of three priming and five booster injections. The primary outcome measures are the CSF levels of anti-α-syn antibodies, assessed approximately six months after the final booster dose. The trial is projected to be completed by April 2025, but no data has been published to date.

### AFFITOPE (PD01A, PD03A), and ACI-7104.056

AFFITOPE PD01A (AFF008) and AFFITOPE PD03A (AFF011) are peptide-based vaccines developed by AFFiRiS company. These active vaccines are designed to generate an immune response against the oligomeric forms of α-syn, aiming to reduce its aggregation and spread in the brain without triggering any deleterious neuroinflammatory responses. PD01A uses a modified eight-amino acid peptide to mimic the C-terminal amino acids of α-syn [15, 16].

A series of Phase I clinical trials of AFFITOPE PD01A (NCT02618941, NCT02216188, NCT01568099, NCT01885494, NCT02758730) have been conducted to evaluate the safety and immunogenicity of PD01A immunisations in patients with PD, followed by three consecutive study extensions. Participants were diagnosed clinically as having PD, which was confirmed using imaging results from dopamine transporter single photon emission computed tomography (DaT-SPECT). A total of 32 patients were recruited, and 24 were deemed eligible. These eligible patients were randomly assigned (1:1) to receive four subcutaneous priming immunisations with either 15 μg or 75 μg of PD01A, administered into the upper arm, with an initial follow-up period of 52 weeks and an additional follow-up of 39 weeks. Subsequently, patients were re-randomised (1:1) to receive a first booster immunisation of either 15 μg or 75 μg with a follow-up period of 24 weeks. All patients then received a second booster immunisation of 75 μg and were followed up for another 52 weeks. Patients remained masked to their dose allocation throughout the study. Of the 24 patients who began the study, one was withdrawn due to a diagnosis change to MSA, and two withdrew consent. Overall, 21 patients completed all six immunisations and remained in the study for 221–259 weeks. Most patients experienced at least one adverse event, although most were unrelated to the study treatment. The most common treatment-related adverse events were transient local injection site reactions, affecting all but one patient. Systemic treatment-related adverse events included fatigue (*n* = 4), headache (*n* = 3), myalgia (*n* = 3), muscle rigidity (*n* = 2), and tremor (*n* = 2). Serial MRI assessments showed no signs of any inflammatory processes [17]. Immunogenicity results indicated that the geometric mean antibody titres against the immunising peptide PD01 increased from 1:46 at baseline to 1:3580 at week 12 in the 15 μg group and from 1:76 to 1:2462 at week 12 in the 75 μg group [17]. A post hoc analysis revealed a 51% reduction in CSF α-syn oligomers, supporting the vaccine’s ability to generate a targeted immune response [17]. In contrast, total α-syn, amyloid-beta (Aβ), total tau, and phosphoTau181 levels remained unchanged [17]. Antibody levels declined to baseline two years after the initial vaccination but rose significantly beyond their original levels following booster doses [17].

A second AFFITOPE antibody, PD03A (NCT02267434), was developed using the same technology as PD01A and underwent a separate Phase I trial. The primary focus was to evaluate safety and tolerability, while a key secondary objective was to examine the immunological response following immunisation. Patients (*n* = 36) were randomised (1:1:1) to receive either PD03A at 15 μg, 75 μg, or a placebo. Each patient underwent four priming vaccinations followed by one booster, with a follow-up period of 52 weeks. Nearly all patients experienced at least one adverse event, with transient local injection site reactions affecting all but two patients [18]. The study demonstrated a robust IgG antibody response against PD03, with the highest titre observed at Week 12 [18]. The differences in antibody titres between active treatment groups (PD03A 15 μg and 75 μg) and the placebo group were statistically significant from the second immunisation at Week 8 until the end of the study at Week 52 [18]. However, PD03A generated lower antibody titres than those observed with PD01A [18].

In 2021, AC Immune acquired the rights to PD01, renaming the optimised formulation ACI-7104.056 (NCT06015841). A Phase II trial began in July 2023 to evaluate the safety, efficacy, and biomarker effects of ACI-7104.056 in early-stage PD patients across Germany, Spain, and the UK. This adaptive-design trial will enrol up to three cohorts, potentially expanding to 150 participants. The primary outcomes include adverse events, MRI changes, and α-syn antibody levels, while secondary outcomes focus on α-syn biomarkers, dopamine transporter scans, and clinical features. As of January 2024, enrolment for the first cohort was completed without safety concerns, and the second cohort was ongoing. The study is expected to conclude in January 2028, providing further insights into the therapeutic potential of ACI-7104.056 in PD. No published data is available yet; only a press release has been released [19].

### Prasinezumab

Prasinezumab is a humanised IgG1 monoclonal antibody targeting aggregated α-syn, developed by Prothena in collaboration with Roche. Preclinical studies suggest that Prasinezumab reduces a neurotoxic, truncated form of α-syn and prevents α-syn propagation between cells, lowering neuropathology and improving behavioural outcomes [15, 20, 21].

The initial Phase I trial (NCT02095171) evaluated safety and pharmacokinetics. The first study involved 40 healthy adults, and Prasinezumab was safe and well-tolerated, reducing free α-syn levels in the blood by up to 96%. A subsequent study (NCT02157714) involved 80 PD patients, which confirmed safety but showed no significant clinical improvement based on the Movement Disorder Society–Unified Parkinsons Disease Rating Scale (MDS-UPDRS). Side effects were mild, including gastrointestinal issues and headaches. The trial indicated that while peripheral α-syn levels dropped, there was no change in CSF α-syn levels [22, 23].

Later, Roche launched the PASADENA Phase II study (NCT03100149) to assess the efficacy of Prasinezumab in 316 newly diagnosed PD patients. Although the trial did not meet its primary efficacy endpoint, the MDS-UPDRS score, there were positive signals in secondary and exploratory outcomes, including a slower decline in motor function as measured by MDS-UPDRS Part III and digital motor assessments. Subsequent analyses indicated more substantial benefits for patients with more severe symptoms or those taking monoamine oxidase-B (MAO-B) inhibitors [24–26].

Roche initiated the PADOVA Phase IIb study (NCT04777331) in May 2021, focusing on patients with early-stage PD. This randomised, double-masked, placebo-controlled, multicenter study enrolled 586 participants who were on stable dopamine replacement therapy. Participants receive monthly intravenous doses of 1500 mg of Prasinezumab or a placebo for at least 76 weeks. After this period, all participants transitioned to a 2 year extension phase where they will receive active treatment. The trial is expected to run until 2024, with an optional two-year open-label extension. No data has been published yet; only an abstract presented at the International Congress of Parkinson’s Disease and Movement Disorders reported that the PADOVA study population is well-suited for assessing the potential of Prasinezumab to slow disease progression in patients with early PD who are on stable symptomatic therapy.

### Cinpanemab

Cinpanemab (BIIB054), a monoclonal antibody developed by Biogen, targets α-syn, binding to residues 1–10 of the N-terminal with 800-fold higher affinity for aggregated over monomeric α-syn. Preclinical studies have demonstrated that Cinpanemab inhibits α-syn spread in cell assays and reduces pathology and motor deficits in mouse models [27].

The Phase I study (NCT02459886) evaluated the safety, tolerability, and pharmacokinetics of BIIB054 through two parts: Part 1**:** A randomised, double-blind, placebo-controlled, single-ascending dose study involving 48 healthy volunteers aged 40–65 years. Participants were assigned to one of six dose groups (1, 5, 15, 45, 90, and 135 mg/kg) to receive a single intravenous infusion of BIIB054 or a saline placebo. Adverse events were comparable across all groups, with common adverse events including headache, dizziness, and procedural pain. Six healthy volunteers experienced treatment-related adverse events such as dysgeusia, diarrhoea, asymptomatic ventricular tachycardia, headache, and hypersensitivity reaction [28]. One serious treatment-related adverse event was reported: a 51-year-old male in the 135 mg/kg group developed an asymptomatic cerebrovascular accident detected on a routine MRI [28]. The patient remained asymptomatic and showed no abnormalities on neurological examination throughout the study. Part 2: Involved 18 participants aged 40–80 years with idiopathic PD, randomly assigned to receive BIIB054 at 15 mg/kg or 45 mg/kg or a placebo. Adverse events were reported in 9 of 12 PD participants receiving BIIB054 and all 6 of those receiving a placebo. Two participants (one on placebo and one on BIIB054 45 mg/kg) experienced treatment-related headaches. One placebo participant had a severe adverse event (grade 3 asthma exacerbation) unrelated to the study treatment [28]. No clinically significant changes were observed in clinical laboratory tests, vital signs, electrocardiograms, physical and neurological examinations, Columbia Suicide Severity Rating Scale, or Montreal Cognitive Assessment in either healthy volunteers or PD participants [28]. Additionally, there were no significant changes from baseline in the MDS-UPDRS, Montreal Cognitive Assessment, or Scale for Outcomes in Parkinson’s Disease for Autonomic Symptoms among participants who received BIIB054. In both healthy volunteers and PD participants, the serum half-life of BIIB054 was 28–35 days, and the cerebrospinal fluid-to-serum ratio ranged from 0.13 to 0.56%. Overall, Phase I results showed that BIIB054 has a favourable safety, tolerability, and pharmacokinetic profile in both healthy volunteers and PD participants, supporting its further clinical development [28].

The SPARK study was a Phase II (NCT03318523); a total of 357 participants were enrolled: 100 in the control group, 55 in the 250 mg Cinpanemab group, 102 in the 1250 mg group, and 100 in the 3500 mg group. At week 52, the changes in MDS-UPDRS scores were 10.8 points for the control group, 10.5 points for the 250 mg group, 11.3 points for the 1250 mg group, and 10.9 points for the 3500 mg group. There were no significant differences between the Cinpanemab and control groups (*P*-values of 0.90, 0.80, and 0.97, respectively). Similar results were observed at week 72, and no significant differences were found in secondary endpoints or DaT-SPECT imaging between any Cinpanemab group and the control. The most common adverse events associated with Cinpanemab were headaches, nasopharyngitis, and falls. Overall, Cinpanemab showed no significant difference compared to placebo in clinical measures of disease progression or changes in DaT-SPECT imaging over a 52 week period in participants with early PD [29]. The trial was discontinued after a week 72 interim analysis due to a lack of efficacy [29].

Another Phase I trial (NCT03716570) was conducted in Japan with 24 PD patients. In February 2021, Biogen stopped Cinpanemab development after the SPARK study failed to meet its primary and secondary endpoints. Both the SPARK and Japanese trials were terminated in April 2021, with published data indicating high variability among participants and no significant biomarker changes with evident clinical progression [30–32].

### MEDI1341

MEDI1341(TAK-341) is a high-affinity monoclonal antibody that targets a C-terminal epitope on both monomeric and aggregated forms of α-syn, initially developed by AstraZeneca. In preclinical studies, MEDI1341 was shown to penetrate the animal brain after intravenous administration, effectively reducing free extracellular α-syn levels in CSF and brain interstitial fluid (ISF). The antibody was shown to prevent the cell-to-cell transmission of human α-syn preformed fibrils (PFFs) in cell cultures and mouse models. In the mouse model of α-syn pathology in the brain, they demonstrated a significant reduction in the accumulation and propagation of α-syn along axons [33].

A phase I trial (NCT03272165) started with single-ascending doses of MEDI1341 in 49 healthy volunteers. Each participant received a 60 min intravenous antibody infusion or a placebo, followed by a three-month observation period. The trial was designed to test up to six different doses, contingent upon safety data. Each dose group consisted of eight volunteers, and the study proceeded to the next higher dose only after the completion of the lower dose cohort. The primary outcome was the monitoring of adverse events. Secondary objectives included analysing pharmacokinetics, measuring α-syn levels in blood and CSF, and identifying anti-drug antibodies in the serum. Another Phase I trial (NCT04449484) investigated multiple ascending doses in 25 participants with PD with intravenous infusions administered every four weeks for a total of three doses. The administration of MEDI1341 or placebo to healthy volunteers and PD did not result in any significant safety concerns across all doses. There were dose-dependent increases in serum concentrations of MEDI1341 across the different groups, and CSF levels confirmed the drug’s penetration into the central nervous system (CNS). In PD, a dose-dependent reduction in free α-syn levels in CSF was observed, consistent with findings from preclinical studies [34, 35].

Later, Takeda took over the development of MEDI1341 and initiated a Phase II trial (NCT05526391) but with MSA patients only.

### Lu AF82422

Lu AF82422 is a humanised monoclonal IgG1 antibody developed by Lundbeck A/S that targets the C-terminal of α-syn. Preclinical studies have demonstrated its safety and CSF target engagement in animal models [36]. Flow cytometry analysis indicated that Lu AF82422 did not bind to the extracellular surface of human platelets, erythrocytes, granulocytes, or lymphocytes. However, it did bind to a small subset of monocytes without affecting their activation or phagocytic function [36].

A Phase I (NCT03611569) single-dose safety study has been done. The study included 59 healthy participants and 15 PD patients. Healthy participants received infusions of one of six doses: 75, 225, 750, 2250, 4500, and 9000 mg or a placebo. PD patients received either 2250 mg or 9000 mg or a placebo. All participants were monitored for 12 weeks. The study also evaluated the pharmacokinetics of this agent in the blood. The treatment was found to be safe and well-tolerated, with no severe adverse events or events leading to withdrawal [37]. The most common treatment-emergent adverse events such as pain at the puncture site, headaches and common infections. Pharmacokinetics were dose-proportional and similar between healthy and PD volunteers. Lu AF82422 plasma levels showed an immediate, concentration-dependent reduction in free α-syn plasma levels and the ratio of free to total α-syn across all cohorts. Also, in the high-dose PD cohort, it lowered the free-to-total α-syn ratio in the CSF [37]. These data supported further development of Lu AF82422.

A Phase II study (NCT05104476), AMULET, is now being planned in patients with MSA only. Additionally, another Phase I trial (NCT06258720) has been registered to assess the safety, tolerability, pharmacokinetics, and immunogenicity of a single infusion of LU AF82422 in healthy Caucasian and Chinese adults. This trial is still ongoing and has a scheduled completion date estimated for September 2024.

These passive α-syn immunotherapies, including Prasinezumab, MEDI1341, and LU AF82422, have been tested for PD in clinical trials. Cinpanemab and ABBV-0805 were withdrawn; ABBV-0805 (NCT04127695) is not included in this review as its development was terminated after a Phase I study.

## Discussion

Targeting α-syn remains a major focus in developing therapeutics for PD. Both active and passive immunotherapy approaches have been studied to reduce α-syn aggregates. Active immunotherapies such as UB-312, AFFITOPE PD01A, PD03A, and ACI-7104.056 are designed to provoke an immune response against α-syn. Meanwhile, passive immunotherapies, including Prasinezumab, Cinpanemab, MEDI1341, and Lu AF82422, directly target α-syn aggregates. Active immunotherapies such as with UB-312 significantly reduce α-syn seeds in the CSF of patients [14]. In addition to post-immunisation analysis from this study, Part A confirmed that IgG fractions and affinity-purified antibodies from healthy volunteers showed strong binding to aggregated α-syn derived from MSA, PD, and recombinant aggregates [14]. However, despite these promising biochemical results, exploratory analyses revealed no statistically significant improvements in clinical scales, highlighting a common challenge in translating biomarker changes into clinical benefits. Applying advanced technologies to improve motor assessments and refine dosing strategies could result in more significant clinical outcomes. Moreover, it remains uncertain whether the antibodies generated after immunisation with UB-312 can penetrate into cells/neurons and effectively target pathological intracellular α-syn [38]. Currently, no definitive in vivo biomarker tools exist to detect α-syn as reliably as postmortem analysis. Objective outcome measures are crucial in future clinical trials for PD, as they offer a more precise and reliable means of tracking disease progression and therapeutic response compared to traditional clinical scales such as the MDS-UPDRS and subjective questionnaires. These objective biomarkers can detect subtle changes in disease state that are often missed by clinical assessments, ultimately improving the evaluation of disease-modifying interventions.

In contrast, Prasinezumab (PASADENA), a passive immunotherapy targeting α-syn, has shown partial efficacy. While the PASADENA trial did not meet its primary endpoint—change from baseline in the MDS-UPDRS (Parts I + II + III)—the study did reveal slower progression of motor features in participants treated with Prasinezumab on the MDS-UPDRS Part III scale [26]. An innovative aspect of the PASADENA trial was using a digital motor score, which combined passive activity monitoring using smartphones and smartwatches with participant performance in smartphone-based activities [26]. This digital approach provided more objective and continuous measures of motor dysfunction in participants’ regular environments, revealing less worsening in participants who received Prasinezumab than those who received placebo [26]. Advanced technology-based assessment tools could enhance our understanding of motor responses and improve the detection of subtle therapeutic effects.

However, not all immunotherapies targeting α-syn have been successful. Cinpanemab, another passive immunotherapy, did not demonstrate significant improvement in motor functions in the Phase II SPARK trial, leading to it not being developed further for PD. SPARK targeted patients with early PD, which is reasonable since advanced PD patients may have lost nearly all of their dopaminergic neurons [39]. For advanced cases, alternative approaches, such as stem cell transplantation or treatments aimed at enhancing the survival of existing cells, may be more appropriate. Despite focusing on early-diagnosed PD, these trials failed to show significant improvements in motor scores. This lack of efficacy may be partially explained by the fact that the correlation between the extent of α-syn pathology, neuronal loss, and clinical features of PD is not fully understood. Even at early stages, if irreversible neuron loss has already occurred, breaking down α-syn aggregates might not lead to substantial motor improvements. As up to 50% of cases could not be classified using the Braak staging system [13], an alternative staging system was developed to address this limitation [40]. Additionally, other studies have reported varied patterns of neuronal loss and dysfunction in early-stage PD [41, 42]. Nonetheless, immunotherapies remain a promising strategy to rescue the remaining vulnerable neurons in PD patients against α-syn pathology.

There are, in theory, a few different scenarios where α-syn pathology can be targeted by a therapeutic antibody. First, due to the cytosolic nature of LB and LN pathology, antibodies could enter the intracellular space to directly neutralise the pathological forms of α-syn, such as oligomers or fibrils. Neutralising these toxic species could reduce their ability to induce cellular dysfunction and promote neurodegeneration. The presence of these antibodies could also interfere with the aggregation kinetics or promote a cell proteostasis response. However, effective permeation of the antibody into the intracellular space is challenging due to the barrier function of the cell membrane and, potentially, the blood-brain barrier, depending on the route of administration, although this field is rapidly advancing technologically[43]. Second, the release of α-syn into the extracellular space, such as the interstitial fluid or the synaptic cleft, maybe a more tractable species to be targeted by therapeutic antibodies. There is ample experimental evidence describing cell-to-cell propagation of α-syn and how such propagation could contribute to the progression of alpha-synucleinopathy [44–46]. There is, nevertheless, no evidence that antibodies can effectively enter the synaptic cleft or penetrate the membrane of extracellular vesicles (e.g. exosomes) to target the α-syn released. Free α-syn may serve as a more plausible extracellular target for antibodies. Recent evidence suggests that local replication, rather than spreading between brain regions, could be the primary mechanism driving the accumulation of Tau aggregates starting from Braak stage III in Alzheimer’s disease (AD) and beyond [47]. Whether the same applies to α-syn aggregation remains to be determined. Finally, similar to the peripheral sink hypothesis originally proposed to support the therapeutic action of anti-amyloid-β antibodies, antibodies that neutralise α-syn in CSF or blood could also lower α-syn concentrations in brain tissue [48]. Such a hypothesis, however, rests on the assumption that α-syn in the brain and outside is in equilibrium. If so, the removal of α-syn in CSF or blood, in conjunction with passive diffusion down a concentration gradient, would lead to a reduction of α-syn in the brain.

Given the absence of definitive disease-modifying therapies for PD, the development of clinical trials targeting α-syn immunotherapy should continue to improve. The primary cause of neuronal cell death in PD remains unknown, with multiple hypotheses, but nothing has yet been confirmed. Given the complexity of PD pathogenesis and its heterogeneity, understanding PD heterogeneity will be essential in subgroups of PD patients and customising treatment approaches. PD is increasingly recognised as a syndrome, encompassing a spectrum of pathophysiological subtypes rather than a single disease entity [49, 50]. This complexity has likely contributed to the limited success of many clinical trials, as uniform therapeutic strategies may not address the distinct biological underpinnings across PD subtypes. Moving forward, a more precise approach, aligned with the principles of personalised medicine, would involve identifying and targeting these subtypes with tailored disease-modifying interventions. In particular, targeting α-syn may be more effective in patients genetically vulnerable to α-syn load, such as those with higher *SNCA* expression. Immunotherapy could be tailored to this subgroup, and a combination of therapies might be more suitable for others. Also, future disease-modifying strategies involve considering the progression of PD and the concept of disease milestones, as discussed by Chen et al. (2024) [51]. Milestone events, such as motor and non-motor symptom transitions, provide key indicators of disease progression and define distinct PD subtypes. Understanding these milestones enables more targeted and effective interventions to modify disease trajectories and improve patient outcomes. In this context, reliable biomarkers play a critical role in tracking disease progression and therapeutic effects. The SAA is a qualitative biomarker to detect pathological protein aggregates like α-syn in PD. However, it does not provide quantitative data on the extent of aggregation or disease progression. As such, it is unsurprising that clinical trials have not shown significant changes using SAA, reinforcing the need for more quantitative biomarkers to better assess therapeutic effects. Similarly, tracking disease progression with the DaT-SPECT scan, as employed in these trials, is not ideal, as it does not necessarily correlate with disease severity or progression.

Additionally, alternative strategies for targeting α-syn are also under investigation, including reducing *SNCA* transcription and translation to lower α-syn expression, as well as using antisense oligonucleotides (ASOs) to target mRNAs to decrease α-syn accumulation [52–55]. Other approaches focus on targeting various genes or proteins associated with alpha-synucleinopathies, such as *glucocerebrosidase*, *LRRK2*, and *poly(ADP-ribose) polymerase-1 (PARP-1*) [56–60],* o*r directly target α-syn aggregation using small molecules like Anle138b [61]. Also, enhancing α-syn clearance is another promising direction, achieved through pathways like autophagy [62–64] and the ubiquitin–proteasome system [65]. Continued research in these areas will be essential for advancing therapeutic strategies for this challenging condition.

Future studies should prioritise the identification of more accurate biomarkers to detect different α-syn species, particularly distinguishing oligomeric from fibrillary forms. Currently, no reliable markers exist specifically for oligomeric α-syn species. Advancements in technology and better biomarkers to distinguish these species, combined with improved measurement tools for PD severity and consideration of PD heterogeneity, could significantly enhance the effectiveness of α-syn-targeted therapies in PD. Finally, more extended follow-up periods are needed in future trials to rule out placebo effects and better understand the long-term impacts of these interventions.

In conclusion, using immunotherapy to target α-syn holds promise as a potential disease-modifying approach for PD. However, the challenges encountered in current immunotherapy clinical trials emphasise the need for further research.

## References

[1] Obeso JA et al (2017) Past, present, and future of Parkinson’s disease: a special essay on the 200th anniversary of the shaking palsy. Mov Disord 32(9):1264–1310. 10.1002/mds.27115 28887905 10.1002/mds.27115 PMC5685546

[2] Stacy M (2011) Nonmotor symptoms in Parkinson’s disease. Int J Neurosci 121(SUPPL. 2):9–17. 10.3109/00207454.2011.620196 22035025 10.3109/00207454.2011.620196

[3] Patterson CG et al (2022) Study in Parkinson’s disease of exercise phase 3 (SPARX3): study protocol for a randomized controlled trial. Trials 23:1. 10.1186/s13063-022-06703-0 36203214 10.1186/s13063-022-06703-0 PMC9535216

[4] Lewy FH (1912) Paralysis agitans. I. pathologische anatomie. Handbuch der Neurologie 3:920–958

[5] Spillantini MG, Schmidt ML, Lee VMY, Trojanowski JQ, Jakes R, Goedert M (1997) α-synuclein in Lewy bodies [8]. Nature 388(6645):839–840. 10.1038/42166 9278044 10.1038/42166

[6] Hasegawa M et al (2002) Phosphorylated α-synuclein is ubiquitinated in α-synucleinopathy lesions. J Biol Chem 277(50):49071–49076. 10.1074/jbc.M208046200 12377775 10.1074/jbc.M208046200

[7] Fujiwara H et al (2002) α-synuclein is phosphorylated in synucleinopathy lesions. Nat Cell Biol 4(2):160–164. 10.1038/ncb748 11813001 10.1038/ncb748

[8] Stefanis L (2012) α-Synuclein in Parkinson’s disease. Cold Spring Harb Perspect Med 2:2. 10.1101/cshperspect.a009399 10.1101/cshperspect.a009399 PMC3281589 22355802

[9] Polymeropoulos MH et al (1979) “Mutation in the α-synuclein gene identified in families with Parkinson’s disease.” Science 276(5321):2045–2047. 10.1126/science.276.5321.2045 10.1126/science.276.5321.2045 9197268

[10] Goedert M, Spillantini MG, Del Tredici K, Braak H (2013) 100 years of Lewy pathology. Nature Rev Neurol. 10.1038/nrneurol.2012.242 10.1038/nrneurol.2012.242 23183883

[11] M. G. Spillantini, R. A. Crowther, R. Jakes, M. Hasegawa, and M. Goedert, “Synuclein in filamentous inclusions of Lewy bodies from Parkinson’s disease and dementia with Lewy bodies (ubiquitinsarkosyl-insoluble filamentsimmunoelectron microscopy),” 1998. [Online]. Available: www.pnas.org. 10.1073/pnas.95.11.6469 PMC27806 9600990

[12] Marti MJ, Tolosa E, Campdelacreu J (2003) Clinical overview of the synucleinopathies. Mov Disord 18(S6):21–27. 10.1002/mds.10559 10.1002/mds.10559 14502652

[13] Braak H, Del Tredici K, Rüb U, De Vos RAI, Jansen Steur ENH, Braak E (2003) Staging of brain pathology related to sporadic Parkinson’s disease. Neurobiol Aging 24(2):197–211. 10.1016/S0197-4580(02)00065-9 12498954 10.1016/s0197-4580(02)00065-9

[14] Eijsvogel P et al (2024) Target engagement and immunogenicity of an active immunotherapeutic targeting pathological α-synuclein: a phase 1 placebo-controlled trial. Nat Med. 10.1038/s41591-024-03101-8 38902546 10.1038/s41591-024-03101-8 PMC11405261

[15] Masliah E et al (2011) Passive immunization reduces behavioral and neuropathological deficits in an alpha-synuclein transgenic model of lewy body disease. PLoS One 6:4. 10.1371/journal.pone.0019338 10.1371/journal.pone.0019338 PMC3084838 21559417

[16] Bae EJ et al (2012) Antibody-aided clearance of extracellular α-synuclein prevents cell-to-cell aggregate transmission. J Neurosci 32(39):13454–13469. 10.1523/JNEUROSCI.1292-12.2012 23015436 10.1523/JNEUROSCI.1292-12.2012 PMC3752153

[17] Volc D et al (2020) Safety and immunogenicity of the α-synuclein active immunotherapeutic PD01A in patients with Parkinson’s disease: a randomised, single-blinded, phase 1 trial. Lancet Neurol 19(7):591–600. 10.1016/S1474-4422(20)30136-8 32562684 10.1016/S1474-4422(20)30136-8

[18] Poewe W et al (2021) Safety and tolerability of active immunotherapy targeting α-synuclein with PD03A in patients with early Parkinson’s disease: a randomized, placebo-controlled, phase 1 study. J Parkinsons Dis 11(3):1079–1089. 10.3233/JPD-212594 34092654 10.3233/JPD-212594 PMC8461711

[19] Andrea Pfeifer, “PRESS RELEASE AC Immune Progress Update on Phase 2 Active Immunotherapy Clinical Pipeline for Alzheimer’s and Parkinson’s diseases,” Jan. 2024. Accessed: Sep. 06, 2024. [Online]. Available: https://ir.acimmune.com/static-files/066e03b5-651e-4862-8084-4194eea0e3bb

[20] Games D et al (2013) Axonopathy in an α-synuclein transgenic model of Lewy body disease is associated with extensive accumulation of c-terminal-truncated α-synuclein. Am J Pathol 182(3):940–953. 10.1016/j.ajpath.2012.11.018 23313024 10.1016/j.ajpath.2012.11.018 PMC3589076

[21] Games D et al (2014) Reducing C-terminal-truncated alpha-synuclein by immunotherapy attenuates neurodegeneration and propagation in Parkinson’s disease-like models. J Neurosci 34(28):9441–9454. 10.1523/JNEUROSCI.5314-13.2014 25009275 10.1523/JNEUROSCI.5314-13.2014 PMC4087215

[22] Schenk DB et al (2017) First-in-human assessment of PRX002, an anti–α-synuclein monoclonal antibody, in healthy volunteers. Mov Disord 32(2):211–218. 10.1002/mds.26878 27886407 10.1002/mds.26878 PMC5324684

[23] Jankovic J et al (2018) Safety and tolerability of multiple ascending doses of PRX002/RG7935, an anti-synuclein monoclonal antibody, in patients with parkinson disease: a randomized clinical trial. JAMA Neurol 75(10):1206–1214. 10.1001/jamaneurol.2018.1487 29913017 10.1001/jamaneurol.2018.1487 PMC6233845

[24] Pagano G et al (2021) A phase II study to evaluate the safety and efficacy of prasinezumab in early Parkinson’s disease (PASADENA): rationale, design, and baseline data. Front Neurol. 10.3389/fneur.2021.705407 34659081 10.3389/fneur.2021.705407 PMC8518716

[25] Pagano G et al (2022) Trial of prasinezumab in early-stage Parkinson’s disease. N Engl J Med 387(5):421–432. 10.1056/nejmoa2202867 35921451 10.1056/NEJMoa2202867

[26] Pagano G et al (2024) Prasinezumab slows motor progression in rapidly progressing early-stage Parkinson’s disease. Nat Med 30(4):1096–1103. 10.1038/s41591-024-02886-y 38622249 10.1038/s41591-024-02886-y PMC11031390

[27] Weihofen A et al (2019) Development of an aggregate-selective, human-derived α-synuclein antibody BIIB054 that ameliorates disease phenotypes in Parkinson’s disease models. Neurobiol Dis 124:276–288. 10.1016/j.nbd.2018.10.016 30381260 10.1016/j.nbd.2018.10.016

[28] Brys M et al (2019) Randomized phase I clinical trial of anti–α-synuclein antibody BIIB054. Mov Disord 34(8):1154–1163. 10.1002/mds.27738 31211448 10.1002/mds.27738 PMC6771554

[29] Kuchimanchi M, Monine M, Kandadi Muralidharan K, Woodward C, Penner N (2020) Phase II dose selection for alpha synuclein-targeting Antibody Cinpanemab (BIIB054) based on target protein binding levels in the brain. CPT: Pharmacomet Syst Pharmacol. 10.1002/psp4.12538 10.1002/psp4.12538 PMC7499191 32613752

[30] Lang AE et al (2022) Trial of cinpanemab in early Parkinson’s disease. N Engl J Med 387(5):408–420. 10.1056/nejmoa2203395 35921450 10.1056/NEJMoa2203395

[31] Hutchison RM et al (2021) Evaluating dopamine transporter imaging as an enrichment biomarker in a phase 2 Parkinson’s disease trial. BMC Neurol 21:1. 10.1186/s12883-021-02470-8 34814867 10.1186/s12883-021-02470-8 PMC8609885

[32] Hutchison RM et al (2024) Cinpanemab in early Parkinson disease. Neurology 102:5. 10.1212/WNL.0000000000209137 10.1212/WNL.0000000000209137 38315945

[33] Schofield DJ et al (2019) Preclinical development of a high affinity α-synuclein antibody, MEDI1341, that can enter the brain, sequester extracellular α-synuclein and attenuate α-synuclein spreading in vivo. Neurobiol Dis. 10.1016/j.nbd.2019.104582 31445162 10.1016/j.nbd.2019.104582

[34] Upcott M, Chaprov KD, Buchman VL (2021) Toward a disease-modifying therapy of alpha-synucleinopathies: new molecules and new approaches came into the limelight. Molecules. 10.3390/molecules26237351 34885933 10.3390/molecules26237351 PMC8658846

[35] Shering C et al (2023) Reducing α-synuclein in human CSF; an evaluation of safety, tolerability, pharmacokinetics and pharmacodynamics of MEDI1341, an α-synuclein-specific antibody, in healthy volunteers and Parkinson’s disease patients (P1–11.007). Neurology. 10.1212/WNL.0000000000202579

[36] Fjord-Larsen L et al (2021) Nonclinical safety evaluation, pharmacokinetics, and target engagement of Lu AF82422, a monoclonal IgG1 antibody against alpha-synuclein in development for treatment of synucleinopathies. MAbs 13:1. 10.1080/19420862.2021.1994690 10.1080/19420862.2021.1994690 PMC8555527 34709986

[37] Buur L et al (2024) Randomized phase I trial of the α-synuclein antibody Lu AF82422. Mov Disord 39(6):936–944. 10.1002/mds.29784 38494847 10.1002/mds.29784

[38] Yu HJ et al (2022) A randomized first-in-human study with UB-312, a UBITh® α-synuclein peptide vaccine. Mov Disord 37(7):1416–1424. 10.1002/mds.29016 35426173 10.1002/mds.29016 PMC9545051

[39] Surmeier DJ, Obeso JA, Halliday GM (2017) Selective neuronal vulnerability in Parkinson disease. Nat Rev Neurosci 18(2):101–113. 10.1038/nrn.2016.178 28104909 10.1038/nrn.2016.178 PMC5564322

[40] Beach TG et al (2009) Unified staging system for Lewy body disorders: Correlation with nigrostriatal degeneration, cognitive impairment and motor dysfunction. Acta Neuropathol 117(6):613–634. 10.1007/s00401-009-0538-8 19399512 10.1007/s00401-009-0538-8 PMC2757320

[41] Jellinger KA (2009) A critical evaluation of current staging of α-synuclein pathology in Lewy body disorders. Biochim Biophys Acta Mol Basis Dis 1792(7):730–740. 10.1016/j.bbadis.2008.07.006 10.1016/j.bbadis.2008.07.006 18718530

[42] Milber JM et al (2012) Parkinson disease and incidental Lewy body disease. Neurology 85(4):1670–1679. 10.1212/WNL.0b013e318278fe32 10.1212/WNL.0000000000002102 PMC4653112 26468408

[43] Slastnikova TA, Ulasov AV, Rosenkranz AA, Sobolev AS (2018) Targeted intracellular delivery of antibodies: the state of the art. Front Pharmacol. 10.3389/fphar.2018.01208 30405420 10.3389/fphar.2018.01208 PMC6207587

[44] Li JY et al (2008) Lewy bodies in grafted neurons in subjects with Parkinson’s disease suggest host-to-graft disease propagation. Nat Med 14(5):501–503. 10.1038/nm1746 18391963 10.1038/nm1746

[45] Kordower JH et al (2011) Transfer of host-derived alpha synuclein to grafted dopaminergic neurons in rat. Neurobiol Dis 43(3):552–557. 10.1016/j.nbd.2011.05.001 21600984 10.1016/j.nbd.2011.05.001 PMC3430516

[46] Kordower JH, Chu Y, Hauser RA, Freeman TB, Olanow CW (2008) Lewy body-like pathology in long-term embryonic nigral transplants in Parkinson’s disease. Nat Med 14(5):504–506. 10.1038/nm1747 18391962 10.1038/nm1747

[47] G. Meisl *et al.*, “In vivo rate-determining steps of tau seed accumulation in Alzheimer’s disease,” 2021. [Online]. Available: https://www.science.org 10.1126/sciadv.abh1448 PMC8555892 34714685

[48] RB DeMattos, KR Bales, DJ Cummins, JC Dodart, SM Paul, DM Holtzman, “Peripheral anti-A antibody alters CNS and plasma A clearance and decreases brain A burden in a mouse model of Alzheimer’s disease,” Jul. 2001. [Online]. Available: 10.1073/pnas.151261398 10.1073/pnas.151261398 PMC37524 11438712

[49] Espay AJ et al (2020) Disease modification and biomarker development in Parkinson disease: revision or reconstruction? Neurology 94(11):481–494. 10.1212/WNL.0000000000009107 32102975 10.1212/WNL.0000000000009107 PMC7220234

[50] Marras C et al (2024) Transitioning from subtyping to precision medicine in Parkinson’s disease: a purpose-driven approach. Mov Disord. 10.1002/mds.29708 38243775 10.1002/mds.29708

[51] Chen S, Wang M-Y, Shao J-Y, Yang H-Q, Zhang H-J, Zhang J-W (2024) Disease progression subtypes of Parkinson’s disease based on milestone events. J Neurol 271(10):6791–6800. 10.1007/s00415-024-12645-1 39187742 10.1007/s00415-024-12645-1

[52] Mittal S et al (1979) 2017, “β2-adrenoreceptor is a regulator of the α-synuclein gene driving risk of Parkinson’s disease.” Science 357(6354):891–898. 10.1126/science.aaf3934 10.1126/science.aaf3934 PMC5761666 28860381

[53] Kantor B et al (2018) Downregulation of SNCA expression by targeted editing of DNA methylation: a potential strategy for precision therapy in PD. Mol Ther 26(11):2638–2649. 10.1016/j.ymthe.2018.08.019 30266652 10.1016/j.ymthe.2018.08.019 PMC6224806

[54] Alarcón-Arís D et al (2020) Anti-α-synuclein ASO delivered to monoamine neurons prevents α-synuclein accumulation in a Parkinson’s disease-like mouse model and in monkeys. EBioMedicine. 10.1016/j.ebiom.2020.102944 32810825 10.1016/j.ebiom.2020.102944 PMC7452525

[55] Cole TA et al (2021) α-Synuclein antisense oligonucleotides as a disease-modifying therapy for Parkinson’s disease. JCI Insight 6:5. 10.1172/jci.insight.135633 10.1172/jci.insight.135633 PMC8021121 33682798

[56] L. F. Burbulla, S. Jeon, J. Zheng, P. Song, R. B. Silverman, and D. Krainc, “Modulator of wild-type glucocerebrosidase improves pathogenic phenotypes in dopaminergic neuronal models of Parkinson’s disease,” 2019. [Online]. Available: https://www.science.org 10.1126/scitranslmed.aau6870 PMC7359409 31619543

[57] Aflaki E et al (2016) A new glucocerebrosidase chaperone reduces α-synuclein and glycolipid levels in iPSC-derived dopaminergic neurons from patients with Gaucher disease and parkinsonism. J Neurosci 36(28):7441–7452. 10.1523/JNEUROSCI.0636-16.2016 27413154 10.1523/JNEUROSCI.0636-16.2016 PMC4945664

[58] Mazzulli JR et al (2016) Activation of β-glucocerebrosidase reduces pathological α-synuclein and restores lysosomal function in Parkinson’s patient midbrain neurons. J Neurosci 36(29):7693–7706. 10.1523/JNEUROSCI.0628-16.2016 27445146 10.1523/JNEUROSCI.0628-16.2016 PMC4951575

[59] Daher JPL et al (2015) Leucine-rich repeat kinase 2 (LRRK2) pharmacological inhibition abates α-synuclein gene-induced neurodegeneration. J Biol Chem 290(32):19433–19444. 10.1074/jbc.M115.660001 26078453 10.1074/jbc.M115.660001 PMC4528108

[60] Kam TI et al (2018) Poly(ADP-ribose) drives pathologic a-synuclein neurodegeneration in Parkinson’s disease. Science 362:6414. 10.1126/science.aat8407 10.1126/science.aat8407 PMC6431793 30385548

[61] Levin J et al (2014) The oligomer modulator anle138b inhibits disease progression in a Parkinson mouse model even with treatment started after disease onset. Acta Neuropathologica. 10.1007/s00401-014-1265-3 24615514 10.1007/s00401-014-1265-3 PMC3984662

[62] Pagan FL et al (2019) Pharmacokinetics and pharmacodynamics of a single dose Nilotinib in individuals with Parkinson’s disease. Pharmacol Res Perspect 7:2. 10.1002/prp2.470 10.1002/prp2.470 PMC6412143 30906562

[63] Simuni T et al (2021) Efficacy of nilotinib in patients with moderately advanced Parkinson disease. JAMA Neurol 78(3):312. 10.1001/jamaneurol.2020.4725 33315105 10.1001/jamaneurol.2020.4725 PMC7737147

[64] Spencer B et al (2009) Beclin 1 gene transfer activates autophagy and ameliorates the neurodegenerative pathology in α-synuclein models of Parkinson’s and Lewy body diseases. J Neurosci 29(43):13578–13588. 10.1523/JNEUROSCI.4390-09.2009 19864570 10.1523/JNEUROSCI.4390-09.2009 PMC2812014

[65] Chatterjee D et al (2018) Proteasome-targeted nanobodies alleviate pathology and functional decline in an α-synuclein-based Parkinson’s disease model. NPJ Parkinsons Dis 4:1. 10.1038/s41531-018-0062-4 30155513 10.1038/s41531-018-0062-4 PMC6105584

